# Reovirus μ2 Protein Impairs Translation to Reduce U5 snRNP Protein Levels

**DOI:** 10.3390/ijms24010727

**Published:** 2022-12-31

**Authors:** Simon Boudreault, Carole-Anne Martineau, Laurence Faucher-Giguère, Sherif Abou-Elela, Guy Lemay, Martin Bisaillon

**Affiliations:** 1 Département de Biochimie et Génomique Fonctionnelle, Faculté de Médecine et des Sciences de la Santé, Université de Sherbrooke, Sherbrooke, QC J1E 4K8, Canada; 2 Département de Microbiologie et Infectiologie, Faculté de Médecine et des Sciences de la Santé, Université de Sherbrooke, Sherbrooke, QC J1E 4K8, Canada; 3 Département de Microbiologie, Infectiologie et Immunologie, Faculté de Médecine, Université de Montréal, Montréal, QC H3C 3J7, Canada

**Keywords:** reovirus, alternative splicing, U5 SNRNP, polysome profiling, translation inhibition

## Abstract

Mammalian orthoreovirus (MRV) is a double-stranded RNA virus from the *Reoviridae* family that infects a large range of mammals, including humans. Recently, studies have shown that MRV alters cellular alternative splicing (AS) during viral infection. The structural protein μ2 appears to be the main determinant of these AS modifications by decreasing the levels of U5 core components EFTUD2, PRPF8, and SNRNP200 during infection. In the present study, we investigated the mechanism by which μ2 exerts this effect on the U5 components. Our results revealed that μ2 has no impact on steady-state mRNA levels, RNA export, and protein stability of these U5 snRNP proteins. However, polysome profiling and metabolic labeling of newly synthesized proteins revealed that μ2 exerts an inhibitory effect on global translation. Moreover, we showed that μ2 mutants unable to accumulate in the nucleus retain most of the ability to reduce PRPF8 protein levels, indicating that the effect of μ2 on U5 snRNP components mainly occurs in the cytoplasm. Finally, co-expression experiments demonstrated that μ2 suppresses the expression of U5 snRNP proteins in a dose-dependent manner, and that the expression of specific U5 snRNP core components have different sensitivities to μ2’s presence. Altogether, these results suggest a novel mechanism by which the μ2 protein reduces the levels of U5 core components through translation inhibition, allowing this viral protein to alter cellular AS during infection.

## 1. Introduction

Mammalian orthoreovirus (MRV) is a double-stranded RNA (dsRNA) virus from the *Reoviridae* family which has a large range of mammalian natural hosts [1]. MRV has a double layered capsid harboring a genome of 10 dsRNA segments which produce eight structural and four non-structural proteins [1]. The structural protein μ2, encoded by the M1 segment, has multiple crucial functions during MRV replication. In the virions, μ2 acts as a cofactor for the RNA-dependent RNA polymerase λ3 and, together, they form the viral transcriptase and replicase complex [2]. Moreover, μ2 harbors both NTPase and RNA triphosphatase enzymatic activities [3]. During the viral replication cycle, μ2 is also involved in the formation and morphology of viral factories, which are cytoplasmic inclusions mainly established by the viral protein μNS and where viral replication takes place [4]. Through its interaction with μNS, μ2 tethers the viral factories to cellular microtubules, which confers a filamentous morphology to viral factories in some reovirus strains [4,5,6]. Moreover, μ2 has been linked to the control of the interferon response [7,8,9], the oncolytic properties of the virus [10,11], cell tropism [12,13], and has been shown to bind RNA [14]. Finally, it was recently shown that μ2 is the main determinant of the cellular alternative splicing (AS) modifications occurring during MRV infection [15,16,17].

AS is a post-transcriptional maturation process of pre-mRNA that allows the production of different mature transcripts from the same gene. This maturation step is crucial to increase the coding potential of the eukaryote genome, as up to 94% of human genes are alternatively spliced, and allows the fine-tuning of the gene products [18,19,20,21,22,23]. AS arises from the spliceosome removing sections of the pre-mRNA that have a coding potential, as opposed to constitutive splicing, where only non-coding portions (i.e., introns) are removed. The spliceosome is a massive ribonucleoprotein complex composed of five small nuclear ribonucleoproteins (snRNP) (i.e., U1, U2, U4, U5 and U6) [24]. snRNP are themselves composed of multiple proteins and one small nuclear RNA [24]. Briefly, splicing starts with intron recognition, both by the U1 snRNP recognizing the 5′ splice site, and the U2 snRNP binding the branch point sequence in close proximity to the 3′ splice site. The A complex recruits the U4/U6. U5 tri-snRNP, forming the B complex. Activation of the B complex requires a drastic rearrangement of the machinery and is mainly driven by U5 snRNP proteins SNRNP200 (helicase) and EFTUD2 (GTPase) and induces the release of the U4 and U1 snRNP. The activated B complex can now catalyze two consecutive trans-esterification reactions leading to the ligation of the exon and the release of the intron in the form of a lariat. U5 snRNP main component PRPF8 is notably crucial for this part of the reaction, as PRPF8 can bind both the 5′ and the 3′ splice-site and maintain the two fragments of the pre-mRNA in close proximity [25]. Spliceosome assembly at different splice sites dictates the sequence identified for removal, and is dictated by the strength of the splice sites, cis-elements in the RNA that can stabilize or destabilize spliceosome assembly, and the respective distance of these cis-regulatory elements from the splice site [26,27].

Some viruses, such as HIV, HPV, and adenovirus, have been known for a long time to usurp the cellular splicing machinery to splice their own genes [28]. However, it was only recently that we began to uncover the drastic impact that viruses have on the cellular AS landscape during infection [29,30,31]. For instance, it was demonstrated that MRV alters AS during the infection of murine L929 cells [15,17]. More recently, it was shown that the viral protein μ2 is the main determinant of these changes in AS [16]. The μ2 protein notably interacts with the three main components of the U5 snRNP, namely EFTUD2, PRPF8, and SNRNP200 [16]. These three proteins are required for MRV modulation of cellular AS, underlining the involvement of the U5 snRNP in this process [16]. During infection, MRV induces a decrease of these three proteins which is likely responsible for the observed modifications in AS [16]. This decrease can be directly linked to the presence of μ2, since its ectopic expression in HEK 293T cells leads to a reduction of PRPF8 and SNRNP200 protein levels [16]. However, the molecular mechanism by which the μ2 protein exerts this effect on U5 snRNP core components is still unknown.

In this study, we sought to determine how μ2 induces a reduction of these U5 snRNP proteins. Our results shed light on a novel impact of μ2 on translation, which suggests this viral protein inhibits translation to reduce the levels of spliceosomal proteins, thereby altering cellular AS during infection.

## 2. Results

### 2.1. The μ2 Protein Does Not Impact EFTUD2, PRPF8, and SNRNP200 mRNA Levels or Nuclear Export

**Figure 1:**
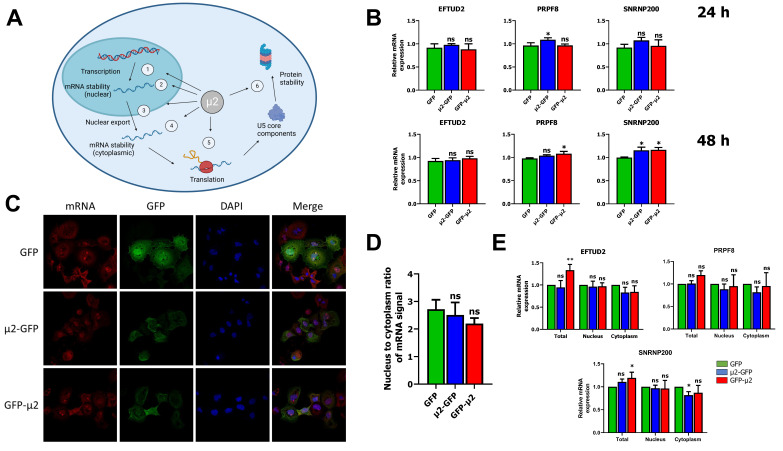
The μ2 protein does not impact EFTUD2, PRPF8, and SNRNP200 mRNA levels or nuclear export: (**A**) outline of the different mechanisms by which the μ2 protein could alter the renewal and proteins levels of U5 snRNP core components. Created with Biorender.com; (**B**) relative mRNA levels determined by qPCR for EFTUD2, PRPF8, and SNRNP200 in GFP or μ2-GFP expressing cells. RNA was harvested with Qiazol at 24 h or 48 h post-transfection, reverse-transcribed, and subjected to qPCR with MRPL19, PUM1, and YWHAZ used as housekeeping genes. The first replicate in the GFP control condition was fixed at 1, and the relative mRNA expression was calculated for all other samples relative to that one. *n* = 3, biological replicates, one-way ANOVA with Dunnett’s multiple comparisons test against the GFP alone condition (ns, *p* > 0.05; *, *p* ≤ 0.05); (**C**) COS-7 cells were transfected for 24 h and mRNA was labelled using a 5’Cy oligo (dT) using standard FISH procedure, as described in Materials and Methods. Nuclei were labelled using DAPI, and GFP signal was imaged at 488 nm; (**D**) quantification of mRNA signal was performed with Cell Profiler. The mRNA signal from cells expressing GFP or μ2-GFP (between 15 and 21 cells from three different fields) was quantified for each condition and is shown. One-way ANOVA with Dunnett’s multiple comparisons test against the GFP alone condition (ns, *p* > 0.05); and (**E**) relative mRNA level determined by qPCR for EFTUD2, PRPF8, and SNRNP200 in total, nuclear, and cytoplasmic fractions from GFP or μ2-GFP expressing cells at 24 h post-transfection. Cells were fractionated, RNA was isolated from total, nuclear, and cytoplasmic fractions with Qiazol, reverse-transcribed and subjected to qPCR with MRPL19, 5.8S, and YWHAZ as housekeeping genes. The relative expression is calculated against the GFP condition for each fraction (total, nuclear, cytoplasmic) in each replicate. *n* = 3, biological replicates, two-way ANOVA with Dunnett’s multiple comparisons test against the GFP alone condition for each fraction (ns, *p* > 0.05; *, *p* ≤ 0.05; **, *p* ≤ 0.01). Results presented are mean ± standard deviation.

As previously described, core components of the U5 snRNP EFTUD2, PRPF8, and SNRNP200 are decreased during infection by MRV [16]. This effect can be recapitulated in transfected HEK 293T cells, in which the ectopic expression of μ2 is sufficient to reduce the levels of PRPF8 and SNRNP200 at 48 h post-transfection [16]. Our previous work suggested that the renewal of these U5 proteins is affected by μ2 [16]. We thus reasoned that μ2 could affect either the transcription, the mRNA export, the mRNA stability (either nuclear or cytoplasmic), the translation of these mRNA or, alternatively, μ2 could enhance the degradation of these proteins in the cytoplasm following translation, but prior to their assembly in the mature U5 snRNP and import in the nucleus (Figure 1A).

As a way to determine if there is any effect of μ2 on transcription and mRNA decay of these U5 snRNP proteins, we first measured steady-state mRNA levels of EFTUD2, PRPF8, and SNRNP200 in HEK 293T cells expressing previously described GFP-tagged μ2 constructs or GFP alone at both 24 h and 48 h post transfection [16]. qPCR results showed either no difference in EFTUD2, PRPF8, and SNRNP200 steady-state mRNA levels upon μ2 expression, or a minor increase (Figure 1B). Thus, there is no decrease in the steady-state mRNA levels for these U5 snRNP components that could explain the decrease at the protein level. Moreover, we also monitored the mRNA level of PRPF6, another component of the U5 snRNP that can interact with μ2 [16], and showed that once again, μ2 does not decrease the steady-state mRNA level for this other U5 component (Supplementary Figure S1). These results indicate that μ2 does not exert its control on the U5 snRNP proteins by diminishing the transcription or the stability of their mRNA.

Next, we investigated the impact of μ2 on mRNA export globally, which would prevent U5 snRNP mRNA to reach the cytoplasm to be translated. Numerous viruses produce proteins with the ability to block mRNA export, notably as a defense mechanism to prevent interferon-stimulated mRNA to reach the cytoplasm and produce antiviral proteins to fight back the virus [32]. First, we performed fluorescent in-situ hybridization (FISH) with fluorescent oligo (dT) in COS-7 cells transfected with GFP-μ2 constructs or GFP alone to visualize mRNA through their poly-A tails. Confocal microscopy revealed no nuclear accumulation of mRNA in μ2-expressing cells compared to cells expressing GFP alone (Figure 1C). Moreover, quantification of mRNA signal for multiple cells and images showed no significant enrichment of the nuclear signal upon μ2 expression (Figure 1D). Additionally, L929 cells infected with MRV do not present any mRNA accumulation in the nucleus, arguing against a global mRNA export blockade triggered by a MRV protein (Supplementary Figure S2). These results demonstrate that μ2 does not impair nuclear mRNA export globally. However, μ2 could be specifically blocking mRNA export for a subset of mRNA of interest, and namely the mRNA producing the U5 snRNP proteins EFTUD2, PRPF8, and SNRNP200. To study the impact of μ2 on the export of EFTUD2, PRPF8, and SNRNP200 mRNA specifically, we used biochemical fractionation to generate cytoplasmic and nuclear fractions from GFP, μ2-GFP, or GFP-μ2 expressing cells. First, we validated that pure cytoplasmic and nuclear fractions were obtained using GAPDH and histone H3 markers (Supplementary Figure S3). Then, the experiment was repeated, RNA was harvested from these fractions and submitted to qPCR to measure relative mRNA levels in the cytoplasm and the nucleus. Our results demonstrate that the expression of μ2 did not lead to any accumulation of EFTUD2, PRPF8, and SNRNP200 mRNA in the nucleus nor any decrease in the cytoplasm at 24 h post-transfection (Figure 1E). Moreover, similar results were obtained at 48 h post-transfection (Supplementary Figure S4). A small but statistically significant increase in the nuclear fraction was observed for EFTUD2 and SNRNP200 with the μ2-GFP construction, but the biological relevance of this result is unclear as there is no change in the cytoplasmic fraction. We also monitored the PRPF6 mRNA and showed very similar results for this other U5 snRNP protein (Supplementary Figure S5). Once again, a significant increase was observed in the nuclear fraction upon μ2 expression, but with no concomitant decrease in the cytoplasmic level. The μ2 protein is thus not globally blocking mRNA export, or specifically blocking the export of U5 snRNP core components’ mRNA and leading to insufficient cytoplasmic mRNA for efficient translation. Taken together, these results demonstrate that μ2′s impact on U5 snRNP proteins is not mediated by transcription, RNA stability, or RNA export.

### 2.2. The μ2 Protein Impairs mRNA Translation

**Figure 2:**
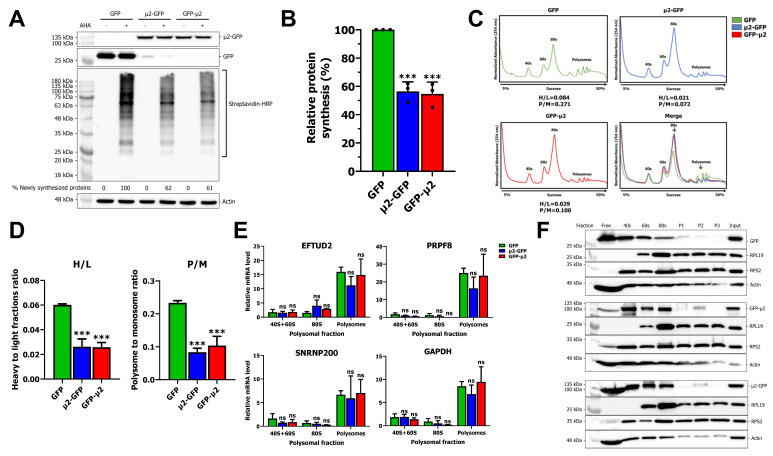
The μ2 protein impairs translation: (**A**) WB of newly synthesized proteins in HEK 293T cells expressing GFP, μ2-GFP, or GFP- μ2. Cells were transfected for 48 h, newly synthesized proteins were metabolically labeled with AHA for 4 h (or DMSO for control), a biotin moiety was added using CLICK chemistry and revealed using streptavidin-HRP. A loading control (actin) was loaded and a WB against GFP was realized to validate the expression of GFP and fusion protein between GFP and μ2; (**B**) quantification of three independent experiments. Biological replicates, *n* = 3, one-way ANOVA with Dunnett’s multiple comparisons test against the GFP alone condition (***, *p* ≤ 0.001); (**C**) polysome profiles for control GFP or μ2-expressing cells. Polysome to monosome ratio (P/M) and heavy to light ratio (H/L) were calculated as described in the material and methods section. Respective profiles were overlapped by aligning the 40S to the same height; (**D**) polysome to monosome ratio (P/M) and heavy to light ratio (H/L) from three independent experiments for control GFP or μ2-expressing cells. Biological replicates, *n* = 3, one-way ANOVA with Dunnett’s multiple comparisons test against the GFP alone condition (***, *p* ≤ 0.001); (**E**) relative mRNA level determined by qPCR for EFTUD2, PRPF8, SNRNP200, and GAPDH in 40S + 60S, 80S, and polysomal (P1 + P2 + P3) fractions from GFP or μ2-GFP expressing cells at 48 h post-transfection. Lysates were prepared, separated on a 5–50% sucrose gradient, and 30% of each fraction was subjected to RNA extraction using Qiazol. RNA was reverse transcribed using a fixed volume for each sample and subjected to qPCR with U6 snRNA as the housekeeping gene, as it was stable between all fractions. The relative expression is calculated against the first sample in the GFP condition for the 40S + 60S fraction. *n* = 3, biological replicates, two-way ANOVA with Dunnett’s multiple comparisons test against the GFP control for each fraction (ns, *p* > 0.05); and (**F**) WB against GFP, RPL19 (large subunit), RPS2 (small subunit), and actin in polysomal fractions from control GFP or μ2-expressing cells at 48 h post-transfection in HEK 293T cells. Results presented are mean ± standard deviation.

Next, we assessed the impact of μ2 on global mRNA translation. Newly synthesized proteins in HEK 293T cells expressing GFP, μ2-GFP, or GFP-μ2 were metabolically labelled with L-azidohomoalanine (AHA) following methionine depletion and detected by CLICK chemistry through the addition of a biotin moiety. Western blot (WB) of newly synthesized proteins revealed a drastic reduction in protein synthesis of approximately 40% upon μ2 expression (Figure 2A). Furthermore, quantification of three independent experiments shows a significant decrease in protein synthesis when μ2 is expressed (Figure 2B). Therefore, we conclude that μ2 impairs global mRNA translation. 

To further analyze the translatomic role of the μ2 protein, polysome profiling was realized on μ2- expressing cells or control cells transfected with GFP alone after 48 h. Polysomes were separated on a sucrose gradient by ultracentrifugation, and fractions were analyzed by spectrophotometry to determine ribosome occupancy on mRNA. Cells expressing μ2 presented a significant reduction in polysomes, and an increase in 80S monosome, as shown by both the polysome to monosome (P/M) ratio and the heavy to light (H/L) ratio (Figure 2C). Moreover, analysis of three independent experiments confirmed a statistically significant reduction in both P/M and H/L ratios upon μ2 expression (Figure 2D). Thus, μ2 impairs translation and induces an accumulation of monosomes, suggesting either a defect in translation initiation, or a decrease in stability of translating ribosomes that become less stable and more prone to fall off rapidly during translation, decreasing the mean occupancy on mRNA. To further understand μ2′s impact on translation, RNA was harvested from these polysomal fractions and subjected to qPCR. As a validation, RNA was run on an automated chip-based microcapillary electrophoresis, which confirmed the efficient fractionation of the polysomal fractions (Supplementary Figure S6). Then, fractions were pooled as such: 40S and 60S; 80S; and P1, P2, and P3, reverse-transcribed and subjected to qPCR for EFTUD2, PRPF8, and SNRNP200. We could not show any statistically significant enrichment of EFTUD2, PRPF8, or SNRNP200 mRNA in the 80S fraction, or reduction of the same mRNA in the polysomal fraction upon μ2 expression (Figure 2E). This result indicates that the impact μ2 exerts on U5 snRNP mRNA is not simply by shifting the mRNA into untranslated fractions and is probably complex. As a control, the GAPDH mRNA also showed no change of enrichment in the different fractions upon μ2 expression (Figure 2E). Other mRNA, such as the SNRPA mRNA from the U1 snRNP and the B2M mRNA were monitored and showed similar result (Supplementary Figure S7). Altogether, these results indicate that μ2 expression induces the accumulation of 80S monoribosome and the decrease of polysomes globally, but no mRNA were specifically shown to shift from polysomes to monosome upon μ2 expression.

Finally, we questioned if the μ2 protein is associated with the untranslated mRNA fraction. WB were performed against the different fractions and revealed that μ2 is mainly associated with the light fractions (free, 40S, 60S, and 80S) (Figure 2F). In contrast, GFP alone elutes mainly in the free fraction, with decreasing levels alongside the increase in sucrose concentration. The actin control also follows the same distribution. In conclusion, μ2 impairs translation by inducing the accumulation of 80S monosomes, decreases the level of mRNA associated with multiribosomes, and associates with light polysomal fractions.

### 2.3. The μ2 Protein Does Not Affect EFTUD2, PRPF8, and SNRNP200 Protein Degradation

**Figure 3:**
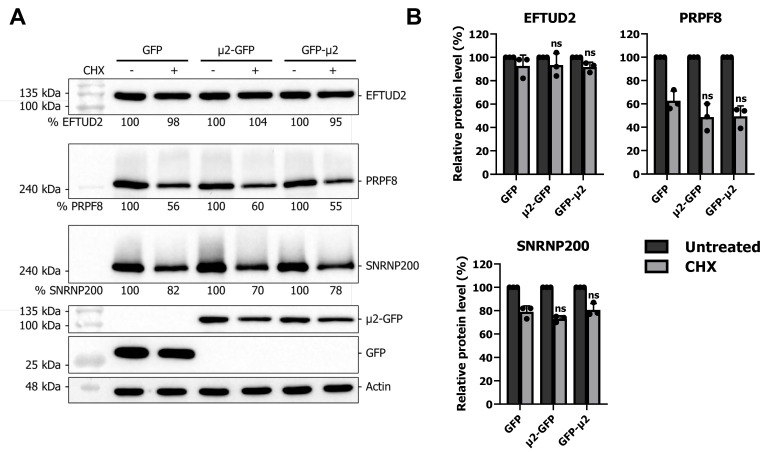
The μ2 protein does not impact EFTUD2, PRPF8, and SNRNP200 protein degradation: (**A**) WB of EFTUD2, PRPF8, and SNRNP200 in HEK 293T cells transfected for 24 h with GFP, μ2-GFP, and GFP-μ2, and then treated with 100 μg/mL CHX or DMSO for an additional 24 h. Relative quantification is shown and normalized for 100% in the same construction for the control condition; and (**B**) quantification of three independent experiments. *n* = 3, biological replicates, two-way ANOVA with Dunnett’s multiple comparisons test against the GFP alone condition; only the result of the comparison in the CHX condition is presented (ns, *p* > 0.05). Results presented are mean ± standard deviation.

To further rule out any implications of other potential mechanisms by which μ2 could decrease U5 proteins levels, we finally tested the impact of μ2 on U5 snRNP protein stability, which would indicate an additional involvement of μ2 in protein degradation. After 24 h of transfection, GFP- or μ2-expressing cells were either treated with DMSO or cycloheximide (CHX) to block translation and incubated for an additional 24 h. Lysate were analyzed for EFTUD2, PRPF8, and SNRNP200 protein levels by WB, and revealed that no increase in EFTUD2, PRPF8, and SNRNP200 degradation could be attributed to the μ2 protein upon CHX treatment (Figure 3A). Quantification of multiple independent experiments confirmed the absence of any effect of μ2 on EFTUD2, PRPF8, and SNRNP200 protein degradation (Figure 3B). These results suggest that the effect of μ2 on U5 snRNP protein levels is not through alteration of protein stability, but is mainly translational, as described in the previous section. 

### 2.4. The Nuclear Localization of μ2 Is Mainly Dispensable for Its Effect on U5 snRNP Protein Levels

**Figure 4:**
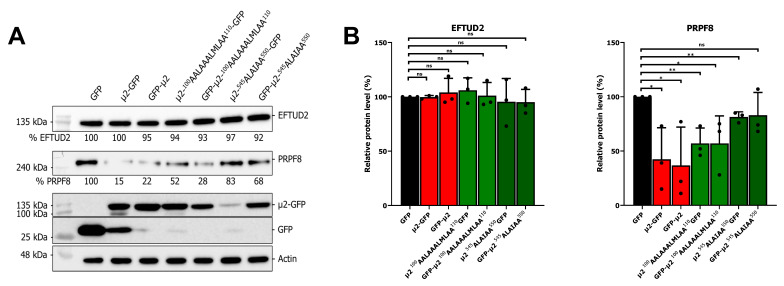
Nuclear localization of μ2 is dispensable for its effect on U5 snRNP protein levels: (**A**) WB of EFTUD2 and PRPF8 upon expression of GFP, μ2-GFP, or mutants of μ2 unable to accumulate in the nucleus for 48 h. Relative quantification is shown in comparison to the GFP alone condition; and (**B**) quantification of three independent WB for EFTUD2 and PRPF8 protein levels. *n* = 3, biological replicates, unpaired two-tailed Student’s *t*-test (ns, *p* > 0.05; *, *p* ≤ 0.05; **, *p* ≤ 0.01) comparing with the GFP condition. Results presented are mean ± standard deviation.

The μ2 protein is localized in both the nucleus and the cytoplasm in transient transfection; however, during infection, it was never observed in the nucleus of infected cells [16,33,34]. Notably, U5 snRNP proteins are mainly nuclear, which suggests that μ2 could interact with these proteins in the nucleus. To assess the necessity of μ2′s presence in the nucleus to reduce U5 snRNP protein levels, we used recently described mutants of μ2 unable to enter the nucleus [16]. Briefly, basic residues in two possible NLS (^100^RRLRKRLMLKK^110^ and ^545^RLKIPY^550^) were mutated to alanine, and these mutations were shown to be sufficient to prevent the nuclear accumulation of μ2 [16]. We monitored the levels of PRPF8, known to be sensitive to μ2’s presence, and EFTUD2, known to be insensitive to the presence of μ2, in cells expressing these mutants tagged with GFP in either N-terminal or C-terminal as well as GFP, μ2-GFP, and GFP-μ2 as described before. The inability of the mutants to enter the nucleus did not drastically impair their ability to decrease the levels of PRPF8 (Figure 4A). This was observed despite the fact that these mutant μ2 proteins are expressed at the same, or slightly decreased level as the wild-type protein. Quantification of three independent experiments revealed that these mutants retain the ability to reduce PRPF8 protein in a similar fashion than the wild-type μ2 (Figure 4B). As previously described, EFTUD2 levels were not changed by the ectopic expression of μ2 [16]. Altogether, these results further suggest that μ2 exert its effect on U5 snRNP proteins in the cytoplasm by blocking mRNA translation, and that the nuclear localization is mainly dispensable for most of this effect.

### 2.5. The Presence of μ2 Hampers the Ectopic Expression of U5 Proteins in a Dose-Dependent Manner

**Figure 5:**
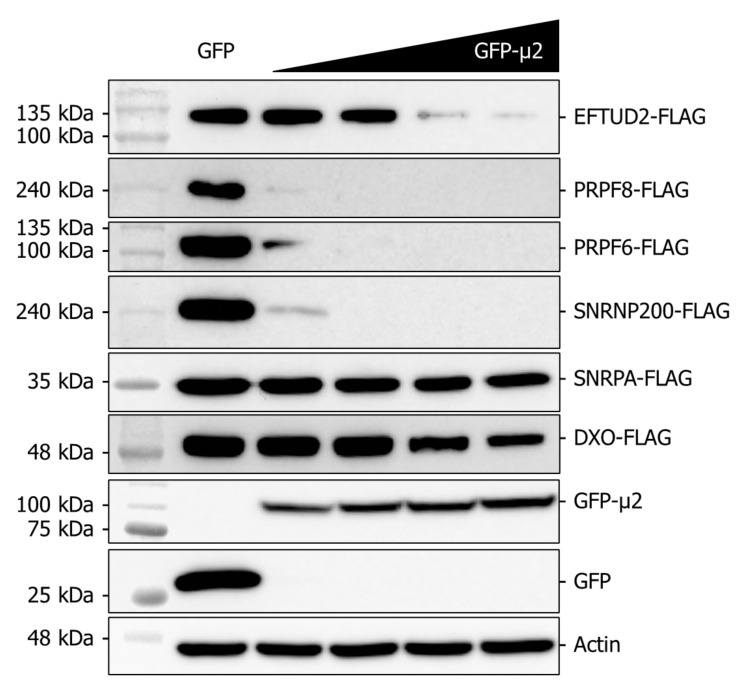
The presence of μ2 hampers the ectopic expression of U5 proteins in a dose-dependent manner. Co-expression of EFTUD2, PRPF8, PRPF6, and SNRNP200 FLAG-tagged constructions with increasing doses of GFP-μ2 for 48 h in HEK 293T cells. SNRPA (U1 snRNP) and DXO (RNA metabolism) were used as controls.

Since U5 snRNP core components are translated in the cytoplasm, but the assembled mature U5 snRNP is nuclear, experiments analyzing the impact of μ2 always monitored EFTUD2, PRPF8, and SNRNP200 without discriminating between the cytoplasmic and the nuclear pools. The fact that μ2 impairs translation in the cytoplasm suggests that the nuclear pools of these proteins are not affected by μ2, and that focusing on the cytoplasmic pool might be more relevant. Moreover, the bulk of these proteins is thought to be localized in the nucleus; this suggests that the effect we observed might be more important and clearer if experiments could be realized in the absence of a pre-existing level of these proteins. To allow the monitoring of the impact of μ2 in the absence of a pre-existing level of these proteins, we tested if the presence of μ2 could suppress the ectopic expression of these U5 snRNP proteins. Moreover, this system also allowed us to investigate if the effect of μ2 on the U5 snRNP proteins is dose-dependent, by progressively increasing the levels of μ2. We therefore co-transfected constant amounts of plasmids encoding EFTUD2-FLAG, PRPF8-FLAG, and SNRNP200-FLAG, alongside increasing amounts of the plasmid encoding GFP-μ2. Even when the lowest amount of GFP-μ2 is expressed, the expression of both PRPF8-FLAG and SNRNP200-FLAG is nearly completely suppressed compared to the GFP control (Figure 5). EFTUD2-FLAG expression is more tolerant to μ2 presence but is also drastically suppressed at higher μ2 concentrations. Moreover, we also tested a PRPF6-FLAG construct, which expression was suppressed by the presence of μ2 in the same fashion as PRPF8-FLAG and SNRNP200-FLAG. As controls, we also monitored another protein of the spliceosome, the U1 snRNP protein SNRPA, which showed a very limited impact of μ2 on SNRPA-FLAG expression. DXO, a decapping enzyme and exonuclease involved in RNA metabolism, was also tested as an additional control, and showed to be much less sensitive to μ2 expression than U5 snRNP proteins. Therefore, we can conclude that U5 proteins expression is highly sensitive by the presence of μ2 in a dose-dependent manner.

## 3. Discussion

In this study, we systemically deciphered the impact of the viral μ2 protein from MRV on transcripts encoding the U5 snRNP proteins EFTUD2, PRPF8, and SNRNP200, in an effort to understand how μ2 triggers a reduction in the level of their corresponding proteins during viral infection [16]. Our data indicate that μ2 does not affect transcription, mRNA export, mRNA stability, and protein stability. In contrast, we could clearly demonstrate that μ2 impairs translation globally, both by monitoring protein synthesis and the polysome profiles in cells expressing μ2 (Figure 2A,C). This is the first report of such an involvement of MRV μ2 protein in translation. The μ2 protein also elutes with light fractions, suggesting it might interact preferentially with untranslated mRNA, or even block mRNA translation (Figure 2F). Notably, the recently published μ2 interactome identified numerous proteins involved in translation as probable μ2 interactors such as eukaryotic initiation factors, notably from the EIF3 complex (*EIF3A*, *EIF3B*, *EIF3C*, *EIF3E*, *EIF3G*, and *EIF4A1*) and components of the large subunit of the ribosome (*RPL3*, *RPL5*, *RPL7A*, *RPL8*, *RPL9*, *RPL10A*, *RPL12*, *RPL28*, *RPL31*, and *RPL35A)* [16]. Moreover, numerous tRNA synthetases, such as *AIPM2*, *EPRS*, *IARS*, *LARS*, *QARS* and *RARS* were also identified as probable μ2 interactors which suggests that μ2 might be involved in aminoacylation of tRNA [16]. Furthermore, an important protein binding to the poly-A tail in the cytoplasm, PABPC1, was also identified as high confidence interactor of μ2 [16]. PABPC1 is notably important during translation, as it interacts both with the poly-A tail and the translation initiation complex, allowing a closed-loop structure and enhancing the translation of polyadenylated mRNAs [35]. Finally, the μ2 interactome is enriched in RNA binding proteins found in stress granules; such granules are believed to be important during MRV infection for the formation of viral factories, and have been linked to the inhibition of translation by sequestering mRNA [36,37,38,39,40,41]. Further studies will be required to decipher the precise mechanism by which μ2 impairs cellular translation, and the potential involvement of the aforementioned proteins and subcellular structures.

**Figure 6:**
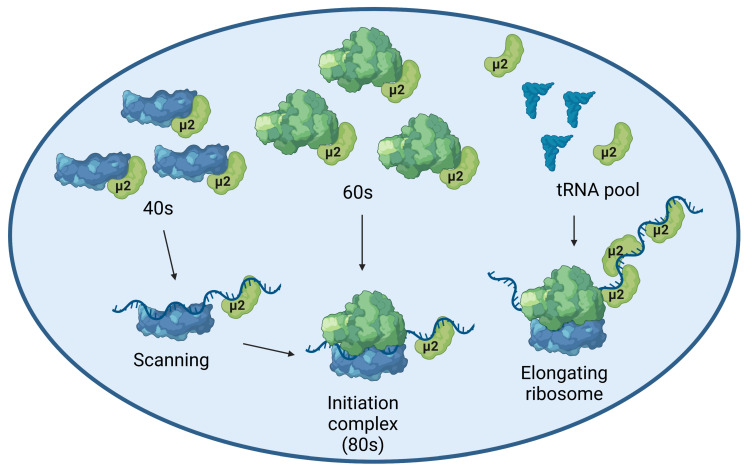
Model depicting how μ2 might exert its effect on translation. μ2 might interact directly with the small subunit of the ribosome, or the large subunit, as recently suggested by IP-MS data of the interactome of μ2 [16]. Moreover, μ2 is bound to cellular mRNA, which might affect the initiation steps of translation. Since μ2 elutes with all the light polysomal fractions (40S, 60S and 80S), all these possibilities are supported by our experimental data. Furthermore, the elongating ribosome is bound to encounter μ2 attached to the mRNA, which might alter the stability of the elongating ribosome. Finally, μ2 might interact with numerous tRNA synthetase, which could affect the pool of loaded tRNA available for the elongating ribosome. Created with Biorender.com.

Moreover, we recently showed that μ2 immunoprecipitates all the mRNA that were tested, suggesting μ2 is physically bound to mRNA, at least in cells where μ2 is ectopically expressed [16]. This suggests that the initiating/elongating ribosome is likely to encounter μ2 on the mRNA during translation, which could either destabilize the ribosome, or induce the release of μ2 from the mRNA. In either case, μ2 is in close proximity to translated mRNA and the ribosome, which suggests its RNA binding activity might be involved in its translational role. A summary of the possibilities of how μ2 exerts its impact on translation is shown in Figure 6. We have also shown that μ2 expression suppresses in a dose-dependent manner the ability of a plasmid to produce U5 snRNP proteins, revealing a specificity of μ2 for these U5 snRNP components (Figure 5). Interestingly, in this experiment, numerous different sizes of mRNA were tested: 7 kb (PRPF8/SNRNP200, 250 kDa), 3 kB (EFTUD2/PRPF6, 135 kDa), and <1.5 kB (SNRPA/DXO, 35–50 kDa). Our results could support the hypothesis that μ2 impairs the translation of the longest mRNA, since controls (SNRPA and DXO) not affected by the μ2 protein are expressed from the smallest mRNA. One interesting possibility would be that μ2 is evenly distributed on the mRNA, and sequential encounters of the ribosome with numerous μ2 proteins increases the likelihood of the ribosome falling off, thus explaining why longer mRNA are more sensitive to μ2′s translational blockade than shorter ones. However, this hypothesis cannot account for the differential impact observed on PRPF6 and EFTUD2, where both the mRNA and the proteins have the same length, while PRPF6 expression is still much more sensitive to μ2′s presence than EFTUD2 (Figure 5). Intriguingly, μ2 expression is not sufficient to shift any mRNA we tested from the heavier to lighter polysomal fractions, even though a clear accumulation of 80S and reduction of polysomal fractions is seen globally. This surprising result suggests the impact of μ2 on translation might be specific to only some mRNA, and could be dictated by other mechanisms, such as direct protein-protein interactions. Notably, we previously demonstrated that μ2 interacts with EFTUD2, PRPF8, and SNRNP200 [16]. This raise the possibility that μ2 could specifically alters the translation of these U5 components by being recruited by the nascent polypeptide during elongation and could alter the remaining elongation or termination. Additional experiments will be required to identify the mechanism by which μ2 alters translation, and the determinants involved in the translational role of μ2. 

MRV has notably been shown to induce a decrease in cellular translation during infection, which differs between different MRV strains, and this decrease has been linked to the viral σ3 protein [42,43,44]. The σ3 protein is a dsRNA binding protein than can prevent the activation of PKR by competing for MRV dsRNA [43]. PKR is an interferon-inducible protein activated by dsRNA; upon activation, PKR can phosphorylate EIF2α, resulting in the inhibition of protein synthesis [45,46]. However, the unclear nature of the 5′ end of MRV mRNA during the course of infection (capped versus non-capped) and the discrepancies in the involvement of PKR in MRV’s inhibition of translation suggest that other viral proteins, such as μ2, might be involved [40,47,48,49,50,51,52,53]. Further studies should be undertaken to decipher, using isogenic viruses, the respective roles of σ3, μ2, and PKR, in cellular translation during MRV infection. 

A potential involvement of μ2 in protein degradation for EFTUD2, PRPF8, and SNRNP200 was also assessed in the current study, and revealed no impact of μ2 on the stability of these U5 snRNP proteins. In these experiments, a 24 h CHX treatment that is not sufficient to reduce the protein levels below 50% and calculate the half-lives of these proteins in HEK 293T cells. This is in agreement with high-throughput experiments assessing protein stability, which calculate the half-life of these U5 snRNP proteins to be between 22 h and 432 h depending on the cell type studied [54]. However, this raises an interesting question: if μ2 only blocks translation, how can it induce such a sharp decrease in PRPF8 (80%), as recently described [16], during the course of a 16 h infection? This suggests that additional mechanisms might be involved beside translation. Moreover, the involvement of other viral proteins might need to be considered and could be investigated. Notably, ectopic expression of the μ2 protein is not able to induce any reduction of EFTUD2 (Figure 4A), whereas during viral infection, a significant reduction of EFTUD2 protein level (close to 25%) was previously shown [16]. Reducing EFTUD2 protein levels might be an indirect effect of the drastic reduction of the other U5 snRNP protein levels, such as PRPF8 and SNRNP200, or could involve another mechanism requiring additional viral proteins. Further studies should aim to clarify this point.

The current study uncovered a novel role for MRV μ2 protein in altering cellular translation. These results suggest μ2, and more broadly MRV, decrease U5 snRNP core components during infection through this translational blockade. Notably, U5 snRNP core components EFTUD2, PRPF8, and SNRNP200, have been linked to key pathways of the antiviral response, in particular apoptosis, necroptosis, and the interferon response during MRV infection [55,56,57,58]. Thus, the translational impact of μ2 has potentially a crucial role in MRV–host interactions, allowing the virus to take advantage on the host cell by reducing EFTUD2, PRPF8, and SNRNP200 protein levels. 

## 4. Materials and Methods

### 4.1. Cells and Reagents

HEK 293T and COS-7 cells were cultured in Dulbecco’s modified Eagle’s medium (DMEM, Wisent) supplemented with 10% fetal bovine serum (Wisent, Saint-Jean Baptiste, QC, Canada). Plasmids encoding the μ2 protein from the T3D^S^ reovirus harboring a GFP moiety (pEGFPN1-μ2, pEGFPC1-μ2) and versions unable to accumulate in the nucleus were described before [1]. The plasmids encoding for EFTUD2 (OHu7961D clone with the human sequence NM_004247.4), PRPF6 (OHu18080D clone with the human sequence NM_012469.4), and SNRPA (OHu15951D clone with the human sequence NM_004596) harboring a C-terminal FLAG were purchased from GenScript, Piscataway, NJ, USA) in the pcDNA3.1-C-(K)DYK backbone and used as supplied. The pcDNA3.1-Hygro MCS-Flag plasmid encoding for SNRNP200 with a N-terminal FLAG was a kind gift of Pr. Daniel Lamarre and Pr. Laurent Chatel-Chaix [2]. The pCMV-HA plasmid encoding the full-length human PRPF8 protein was kindly provided by Pr. Shin-Ru Shih [3]. PRPF8 was subcloned from pCMV-HA to pcDNA3.1- by digesting with NotI and XhoI. A N-terminal FLAG was added by KLD (New England Biolabs, Ipswich, MA, USA) directly in frame with PRPF8 in this plasmid. The pcDNA3.1+ plasmid harboring the human DXO protein was described before [4]. A version harboring a C-terminal FLAG was cloned using the same experimental workflow by introducing the FLAG sequence in the reverse primer. Cycloheximide (CHX) stock solution was prepared at 10 mM.

### 4.2. Plasmid Transfection

HEK 293T cells were plated in a 12-well plate (4 × 10^5^ cells per well), 6-well plate (1 × 10^6^ cells per well for a 24 h transfection; 5 × 10^5^ cells per well for a 48 h transfection) or P100 dish (4 × 10^6^ cells). The next morning, 1 µg (12-well plate), 2.5 µg (6-well plate) or 15 µg (P100 dish) were transfected into the cells using Lipofectamine 2000 following the manufacturer’s protocol. For mRNA FISH, COS-7 cells were plated in a 24-well plate at 1.5 × 10^4^ cells/well on glass coverslips. The next morning, 500 ng of DNA was transfected into the cells using Lipofectamine LTX as recommended by the manufacturer. pEGFPN1 was used to express GFP alone, and 20 times less of this plasmid was transfected to match the GFP expression to the levels of μ2-GFP and GFP-μ2; the empty pcDNA3.1+ vector was used to complete the remaining of plasmid DNA transfected.

### 4.3. RNA Extraction, Reverse Transcription and qPCR

Total RNA samples were extracted with Qiazol^®^ as recommended by the manufacturer (Qiagen, Hilden, Germany). Reverse transcription was performed on 2.2 µg total RNA with Transcriptor reverse transcriptase, random hexamers, dNTPs (Roche Diagnostics), and 10 units of RNAse OUT (Invitrogen) following the manufacturer’s protocol in a total volume of 20 µL. For polysomal fractions, a fixed volume of RNA was used to allow the adequate comparison between different polysomal fractions. For qPCR, all forward and reverse primers were individually resuspended to 20–100 μM stock solution in Tris-EDTA buffer and diluted as a primer pair to 1 μM in RNase DNase-free water (IDT). The complete list of primers used in this study is available in Supplementary Table S1. Quantitative PCR (qPCR) reactions were performed in 10 µL in 96 well plates on a CFX-96 thermocycler (BioRad, Hercules, CA, USA) with 5 μL of 2X iTaq Universal SYBR Green Supermix (BioRad, Hercules, CA, USA), 10 ng (3 µL) cDNA, and 200 nM final (2 µL) primer pair solutions. Once again, for polysomal fraction, a fixed volume of cDNA was used. The following cycling conditions were used: 3 min at 95 °C; 50 cycles: 15 s at 95 °C, 30 s at 60 °C, 30 s at 72 °C. Relative expression levels were calculated using the qBASE framework using 5.8S, MRPL19, PUM1, YWHAZ and U6 snRNA as housekeeping genes. For all PCR runs, control reactions performed in the absence of template were performed for each primer pair, and these were consistently negative. All qPCR data were generated following the MIQE guidelines [59].

### 4.4. Fluorescence In Situ Hybridization (FISH) of mRNA

Cells were transfected for 24 h, then washed with PBS and fixed in 3% paraformaldehyde (in PBS) for 15 min. Cells were rinsed with PBS, permeabilized with 0.2% Triton-X-100 (in PBS) for 30 min, and blocked with in-situ hybridization buffer (2X SSC, 20% formamide, 0.2%, BSA, 1 µg/mL Baker’s yeast tRNA in PBS) for 15 min at 46 °C. The 5′-Cy5 oligo (dT) probe was added to the hybridization buffer at a final concentration of 10 pmol/µL and hybridized at 46 °C overnight. The oligo (dT) probe is composed of 40 thymidine labelled in 5′ with a Cy5 (IDT). The following morning, four washes were performed in the following buffer: 2X SSC, 20% formamide in water for 5 min at 37 °C; 2X SSC in water for 5 min at 37 °C; 1X SSC in water for 5 min at 25 °C and PBS for 5 min at 25 °C. DAPI (1: 10,000 in PBS) was used to stains nuclei at room temperature for 15 min. Cells were then washed two times with PBS and mounted on a slide with SlowFade Diamond mounting medium (Life Technologies, Carlsbad, CA, USA). Images were captured with a Zeiss LSM 880 2 photons confocal microscope.

### 4.5. Cell Fractionation

Cell fractionation was performed using NE-PER™ Nuclear and Cytoplasmic Extraction Reagents (ThermoFisher Scientific, Waltham, MA, USA) as per the manufacturer’s protocol, using HEK 293T cells harvested by trypsin digestion from a 6-well plate.

### 4.6. Newly Synthesized Proteins Assay

This assay was performed as described before [60]. Briefly, 48 h post-transfection, HEK 293T were depleted from methionine by two successive washes with methionine-free DMEM (Wisent, Saint-Jean Baptiste, QC, Canada) and incubated in this medium for 1 h. Newly synthesized proteins were labelled by incubation of cells with L-azidohomoalanine (AHA) to a final concentration of 25 µM for 4 h. Cells were lysed in RIPA buffer (1% Triton X-100, 1% sodium deoxycholate, 0.1% SDS, 1 mM EDTA, 50 mM Tris-HCl pH 7.5 and complete protease inhibitor (Roche, Bâle, Switzerland), sonicated, centrifuged, and the supernatant’s protein content was measured by a Bradford assay. 25 μg of protein was clicked to add a biotin moiety to the newly synthesized proteins. The CLICK reaction was performed by successive addition of the following reagents and vortexing: 0.3 μL of biotin-alkyne (4 mM), 6 μL of CuSO_4_ (20 mM), 3 μL of sodium ascorbate (300 mM), and 3 μL of THPTA (100 mM). A 30 min incubation at room temperature was carried out in the dark and the reaction was stopped by addition of Laemmli sample buffer. For each condition, 15 μg of proteins were loaded on a 10% acrylamide gel for WB.

### 4.7. Western Blot (WB)

Cells were washed with PBS and then lysed in RIPA buffer (1% Triton X-100, 1% sodium deoxycholate, 0.1% SDS, 1 mM EDTA, 50 mM Tris-HCl pH 7.5 and complete protease inhibitor (Roche, Bâle, Switzerland). Samples were sonicated at 13% amplitude for 5 s, two to four times, on ice and insoluble debris were pelleted by a 10 min centrifugation at 13,400 rpm at 4 °C. The supernatant’s protein content measured by a standard Bradford assay (Thermo Scientific Coomassie Protein Assay), Laemmli buffer 4X (200 mM Tris-HCl pH 6.8, 40% glycerol, 1.47 M β-mercaptoethanol, 4% SDS, 0.4 g/L bromophenol blue) was added to the samples to a final concentration of 1X and heated at 95 °C for 5 min. Samples were loaded on a SDS-polyacrylamide gel and migrated at 150 V. Proteins were transferred on a PVDF membrane at 100 V, 4 °C, for 1 h and 15 min. The membranes were blocked in 5% non-fat milk diluted in TBS-T (10 mM Tris-HCl pH 8.0, 220 mM NaCl, 0.1% Tween20). Membranes were incubated overnight at 4 °C in 2.5% non-fat milk diluted in PBS with the primary antibody. The antibodies used in this study are the following: Actin (Sigma, A5441, 1:10,000), EFTUD2 (Abcam, ab188327, 1:2000), FLAG (Sigma, F1804, 1:1000), GFP (Santa Cruz Biotechnology, sc-9996, 1:8000), PRPF8 (Abcam, ab79237, 1:1000), Streptavidin-HRP (ThermoFisher Scientific, N100, 1:5000), RPL19 (Santa Cruz Biotechnology, 1:1000), RPS2 (1:2000, a kind gift from the Mark Bedford lab), Vinculin (Santa Cruz Biotechnology, sc-73614, 1:1000). The next morning, three 5 min washes in TBS-T were carried out. Streptavidin-HRP membranes do not require a secondary antibody and were thus washed once in PBS and revealed. Other membranes were incubated with a secondary antibody (horse anti mouse-HRP secondary antibody (1:5000, Cell Signaling Technologies, 7076) or goat anti rabbit-HRP secondary antibody (1:10,000, Abcam, ab205718) in 2.5% non-fat milk diluted in PBS during 90 min at room temperature. Membranes were washed three times in TBS-T and once in PBS before revelation using Clarity ECL WB substrates (BIORAD); images were acquired on an ImageQuant LAS4000 (GE Healthcare, Chicago, IL, USA). For quantification, HRP was inactivated using 30% H_2_O_2_ for 30 min, followed by two washes in PBS; membranes were then blocked again and probed for the relevant loading control. All WB were performed three times, and a representative result is presented. 

### 4.8. Polysome Profiling

Two P100 dishes were seeded and transfected for each condition; 48 h after transfection, cells were treated with 100 μg/mL of cycloheximide (CHX) for 15 min, washed twice in PBS + 100 μg/mL CHX, and recovered by scraping in the same buffer. Upon centrifugation, the cells from the two dishes were pooled and lysed in 10 mM Tris-HCL pH 7.5, 10 mM NaCl, 1.5 mM MgCl_2_, 1.25% Triton-X-100, 2.5% Tween20, 0.5% deoxycholate, 1X complete protease inhibitor (ROCHE), 1 mM PMSF, 200 units/mL RNAse inhibitor and 100 μg/mL CHX, incubated on ice 10 min, and centrifuged at 13 4000 rpm, 10 min at 4 °C. The supernatants were quantified by Bradford, and between 3 to 4 mg of proteins were loaded on a 5–50% sucrose gradient prepared with 20 mM Tris–HCl pH 8, 140 mM KCl, 5 mM MgCl_2_, 0.5 mM DTT, 100 μg/mL CHX, 0.5 mg/mL heparin. Gradients were centrifuged for 3 h at 4 °C, 40,000 RPM in a SW41 rotor (Beckman Coulter). Fractions were harvested by upward displacement with 55% (*w/v*) sucrose using a gradient fractionator (Brandel Inc., Gaithersburg, MD, USA) connected to a UA-6 UV monitor (Teledyne Isco) for the measurement of 254 nm absorbance. Fractions were collected as follow: Free, 40S, 60S, monosome (80S), P1 (2–3 ribosomes), P2 (4–5–6 ribosomes), and P3 (7+ ribosomes) (Supplementary Figure S8). Then, 30% of each fraction was used to harvest RNA using standard Qiazol extraction while 30% of each fraction was also used to precipitate proteins for WB experiments using a standard DOC-TCA precipitation protocol [61]. Protein precipitates were resuspended in 40 μL of Laemmli 1X sample buffer, and the total volume was loaded on a 10% acrylamide gel. To calculate heavy to light ratios (H/L) and polysome to monosome ratios (P/M), the following equations were used:

$$\frac{H}{L}=\frac{P2+P3}{40S+60S+80S}\;\;\;\;\;\;\;\;\;\;\;\;\;\;\;\;\;\frac{P}{M}=\frac{P1+P2+P3}{80S}\;$$

### 4.9. Protein Degradation Assay

HEK 293T cells were transfected for 24 h, and then treated with DMSO or CHX at 100 μg/mL for an additional 24 h. Lysates were harvested as previously described and subjected to immunoblotting.

### 4.10. Co-Expression Experiments

HEK 293T cells were plated in a 12-well plate at 400,000 cells/well and transfected on the following morning using Lipofectamine2000 (ThermoFisher, Waltham, MA, USA) and 1.5 μg of total plasmid DNA. An aliquot of 500 ng was dedicated to the FLAG-tagged protein plasmid, and the remaining 1000 ng was split between the pEGFPC1-μ2 plasmid and empty pcDNA3.1+ plasmid to make sure every transfection contains the same amount of plasmids. The quantity of pEGFPC1-μ2 plasmid transfected were as follow: 100 ng, 250 ng, 500 ng, and 1000 ng, corresponding to ratio with the FLAG-tagged protein plasmid of 5:1, 2:1, 1:1 and 1:2, respectively. The control pEGFPN1 empty vector to express GFP was transfected at 50 ng alongside 950 ng of empty pcDNA3.1+, to match the GFP alone expression to the highest expression of the GFP-μ2 construction. Cells were harvested 48 h post-transfection.

### 4.11. Data Analysis and Statistical Analyses

Quantification of the mRNA signal in cells expressing μ2-GFP, GFP-μ2, or GFP in the FISH experiment was done using Cell Profiler. Quantification of the protein signals in WB, and integration of the area under the curve for the polysomal profiles, were done with ImageJ. Graphics and statistical tests were realized with GraphPad Prism version 9.4.1. All results presented in this article are mean ± standard deviation.

## Supplementary Materials

The following supporting information can be downloaded at: https://www.mdpi.com/article/10.3390/ijms24010727/s1.
