# Supplementary material for: Reovirus μ2 Protein Impairs Translation to Reduce U5 snRNP Protein Levels
Source: Int J Mol Sci. 2022 Dec 31;24(1):727. doi: 10.3390/ijms24010727 (PMC9821451; doi:10.3390/ijms24010727)
Supplement: Supplementary file 1 [file ijms-24-00727-s001.zip › ijms-2114475-supplementary.pdf]

Supplementary Tables and Figures

Reovirus µ2 protein impairs translation to reduce U5 snRNP protein levels

Simon Boudreault, Carole-Anne Martineau, Laurence Faucher-Giguère, Sherif Abou-Elela, Guy Lemay and Martin Bisaillon

Table S1. qPCR primers

Target	Housekeeping gene	Primers	
		Fw	Rv
B2M		5′-ACTACACTGAATTCACCCCCACTGA-3′	5′-GCTGCTTACATGTCTCGATCCCA-′3
EFTUD2		5′-AGATCATGACGATGACCACCCT-3′	5′-CGGCTGTTGGGTAGTACTTCTTGT-3′
PRPF6		5′-GCAGCCCTGGATAAAAGGATGG-3′	5′-GGATTTTGGGGCGTTCCATACG-3′
PRPF8		5′-GCCCTCTAGCCCCGCTACC-3′	5′-TCCACAAACCCAAACTTCCGCT-3′
RNU2-1		5′-TTTGGCTAAGATCAAGTGTAG-3′	5′-AGCTCCTATTCCATCTCCCT-3′
SNRNP200		5′-TACCTGCATGAGGGGCTCAG-3′	5′-AGCAGAGACTCCGAGAAGCCA-3′
5.8S	✓	5′-ACTCTTAGCGGTGGATCACTCGG-3′	5′-CAAGTGCGTTCGAAGTGTCGATG-3′
U6 snRNA	✓	5′-GCTCGCTTCGGCAGCACATA-3′	5′-ACGCTTCACGAATTTGCGTGTC-3′
MRPL19	✓	5′-AAGGAGAAAAGTACTCCACATTCCAGAG-3′	5′-TGGGTCAGCTGTAGTAACACGA-3′
PUM1	✓	5′-TGAGGTGTGCACCATGAAC-3′	5′-CAGAATGTGCTTGCCATAGG-3′
YWHAZ	✓	5′-TCCCCAATGCTTCACAAGCAGA-3′	5′-TCTTGTCATCACCAGCGGCAA-3′

Figure S1. Relative mRNA levels for PRPF6 in GFP or μ2-GFP expressing cells at 24 h and 48 h post-transfection. RNA was harvested with Qiazol, reverse-transcribed, and subjected to qPCR using a primer pair against PRPF6 with MRPL19, PUM1, and YWHAZ used as housekeeping genes. The first replicate in the GFP control condition was fixed at 1, and the relative mRNA expression was calculated for all other samples relative to that one. n=3, biological replicates, one-way ANOVA with Dunnett's multiple comparisons test against the GFP alone condition (ns, $P>0.05$; **, $P\leq0.01$)

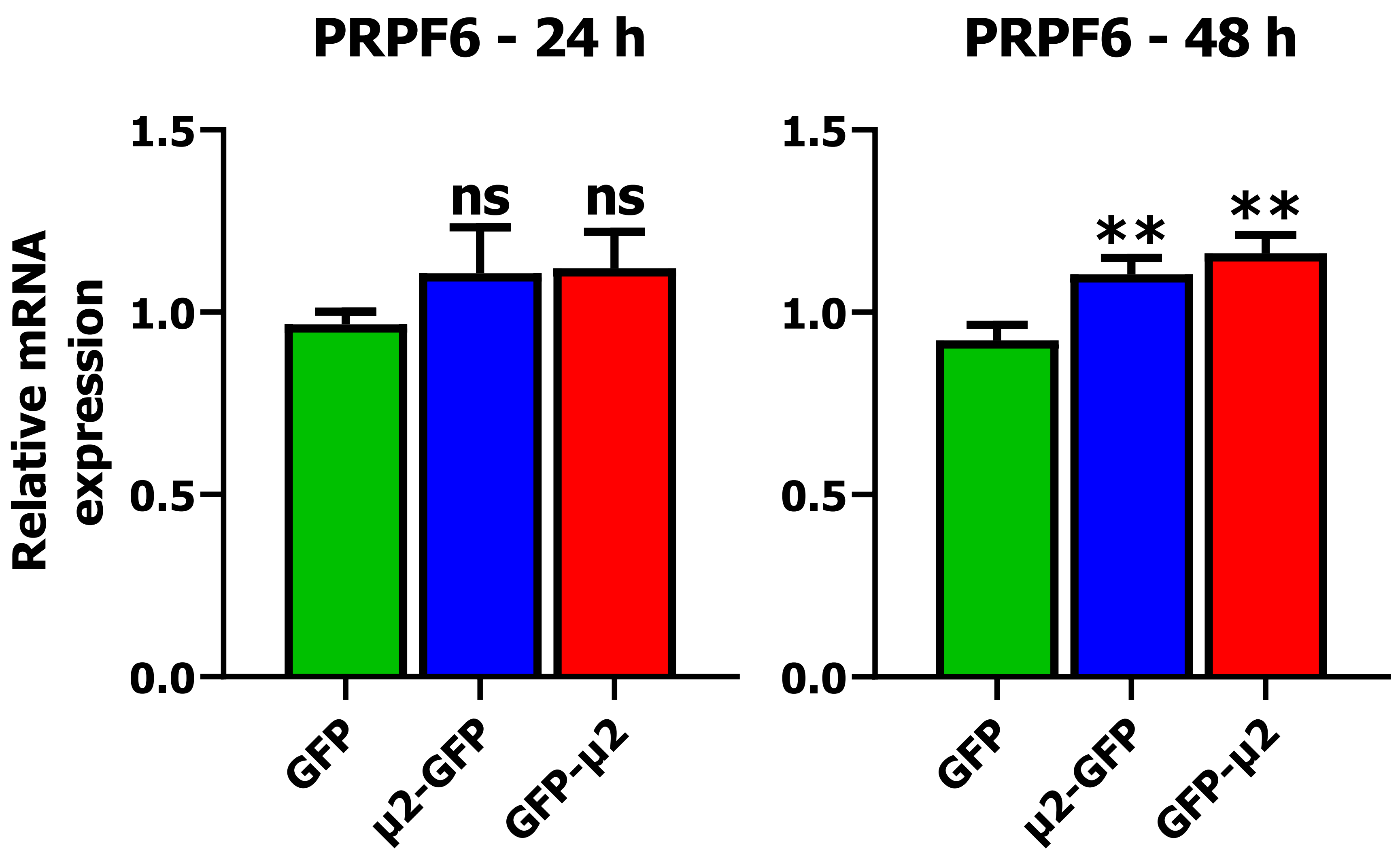
PRPF6 - 24 h
PRPF6 - 48 h
Relative mRNA expression
1.5
1.0
0.5
0.0
ns
ns
**
**
GFP
μ2-GFP
GFP-μ2

Figure S1

Figure S2. mRNA FISH on mock-infected and MRV-infected L929 cells. L929 cells were mock-infected or infected with MRV (T3D^{S} strain) at a MOI of 50, and 16 h post-infection, mRNA were visualized using an oligo (dT) probe labelled with a Cy5 in 5′ using standard FISH procedures. Nuclei were labelled using DAPI.

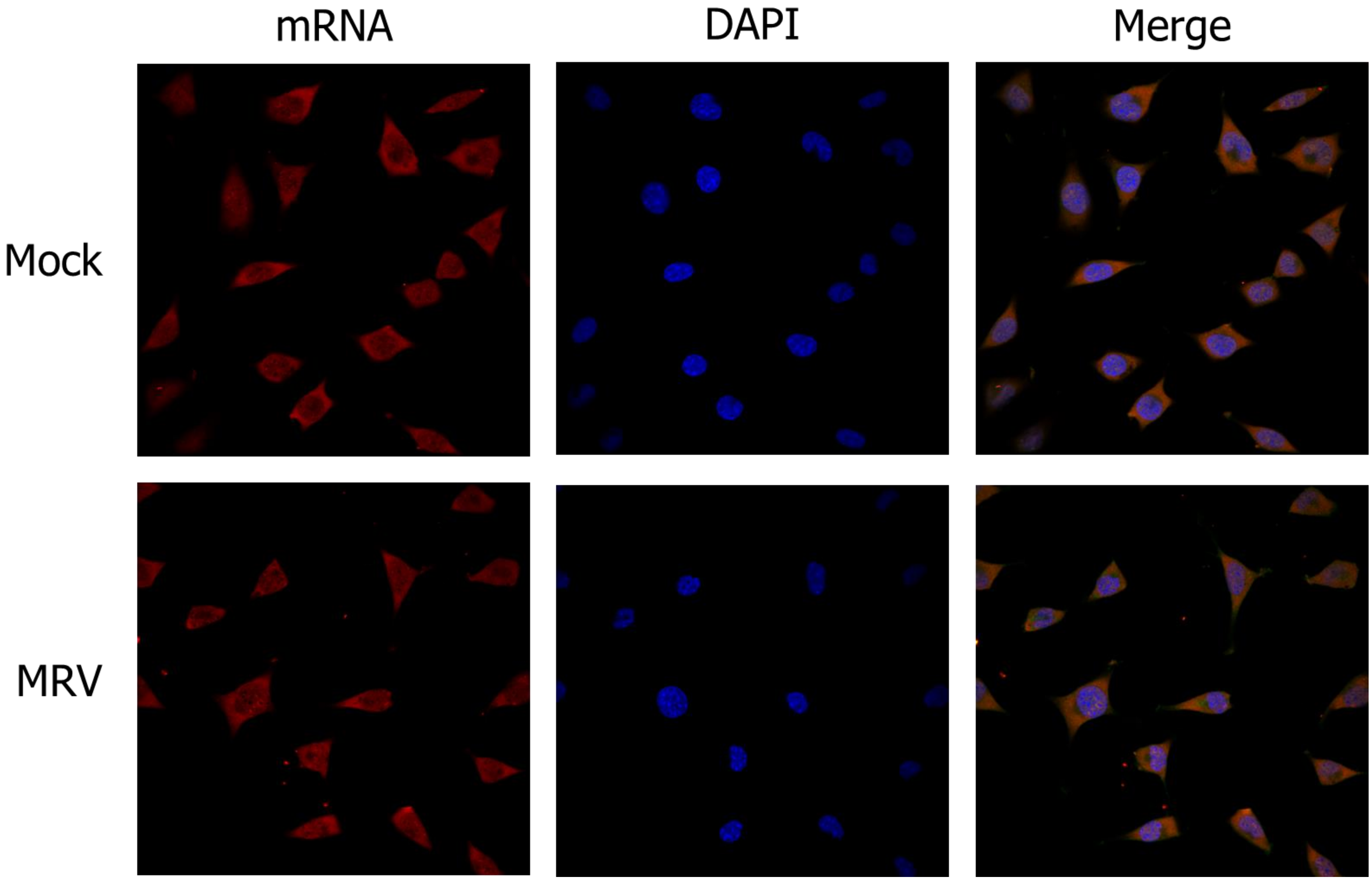

Figure S2

Figure S3. Nuclear and cytoplasmic controls for the subcellular fractionation. Cells were transfected with GFP for 24h, fractionated, and total (T), cytoplasmic (C), and nuclear (N) fractions were subjected to WB. Histone H3 was used as a nuclear marker and GAPDH a cytoplasmic marker.

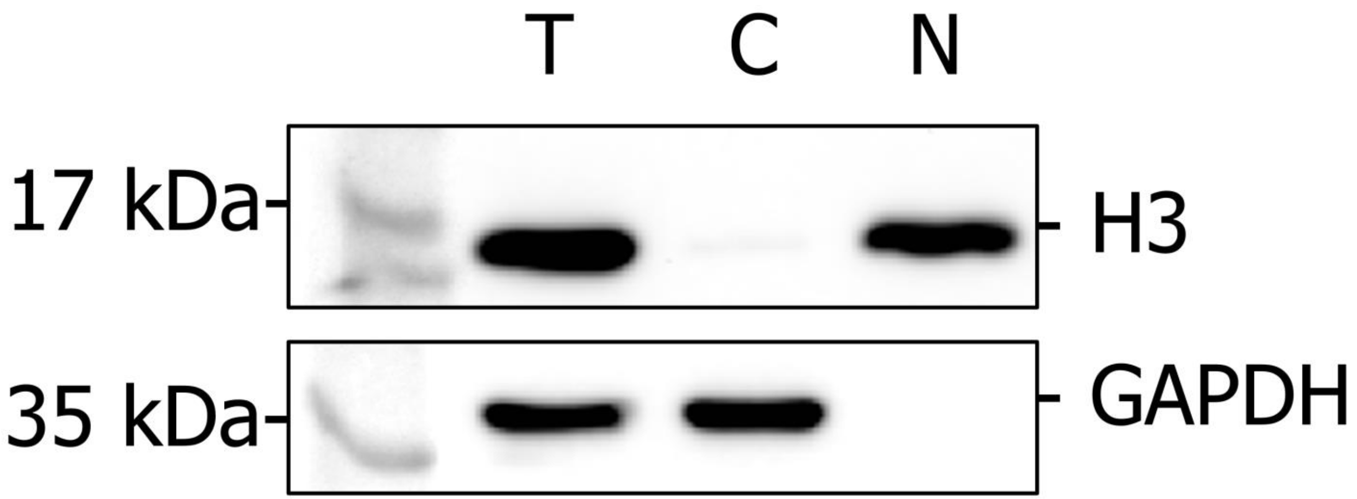

Figure S3

Figure S4. Relative mRNA level for EFTUD2, PRPF8 and SNRNP200 in total, nuclear, and cytoplasmic fractions from GFP or µ2-GFP expressing cells at 48 h post-transfection. Cells were transfected for 48 h, fractionated, RNA was isolated from total, nuclear, and cytoplasmic fractions with Qiazol and reverse-transcribed. qPCR was performed with primer pairs for EFTUD2, PRPF8, SNRNP200, and MRPL19, 5.8S, and YWHAZ as housekeeping genes. Relative expression is calculated against the GFP condition for each fraction (total, nuclear, cytoplasmic). n=3, biological replicates, two-way ANOVA with Dunnett's multiple comparisons test against the GFP alone condition for each fraction (ns, $P>0.05$; *, $P\leq 0.05$)

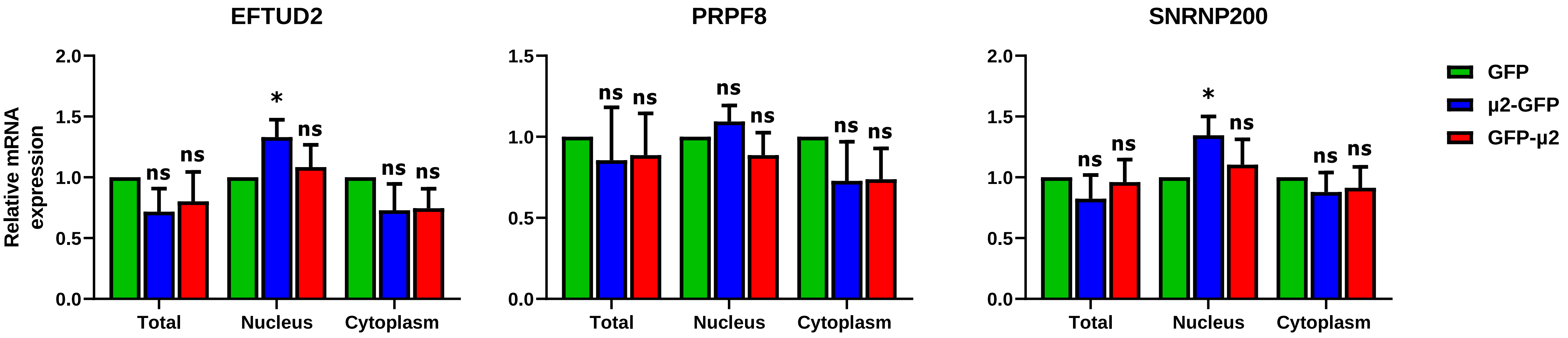
EFTUD2
PRPF8
SNRNP200
Relative mRNA expression
2.0
1.5
1.0
0.5
0.0
*
ns
Total
Nucleus
Cytoplasm
GFP
μ2-GFP
GFP-μ2

Figure S4

Figure S5. Relative mRNA level for PRPF6 in total, nuclear, and cytoplasmic fractions from GFP or μ2-GFP expressing cells. Cells were transfected for 24 h or 48 h, fractionated, RNA was isolated from total, nuclear, and cytoplasmic fractions with Qiazol and reverse-transcribed. qPCR was performed with primer pairs for PRPF6, and MRPL19, 5.8S, and YWHAZ as housekeeping genes. Relative expression is calculated against the GFP condition for each fraction (total, nuclear, cytoplasmic). n=3, biological replicates, two-way ANOVA with Dunnett's multiple comparisons test against the GFP alone condition for each fraction (ns, P>0.05; *, P≤0.05; **, P≤0.01

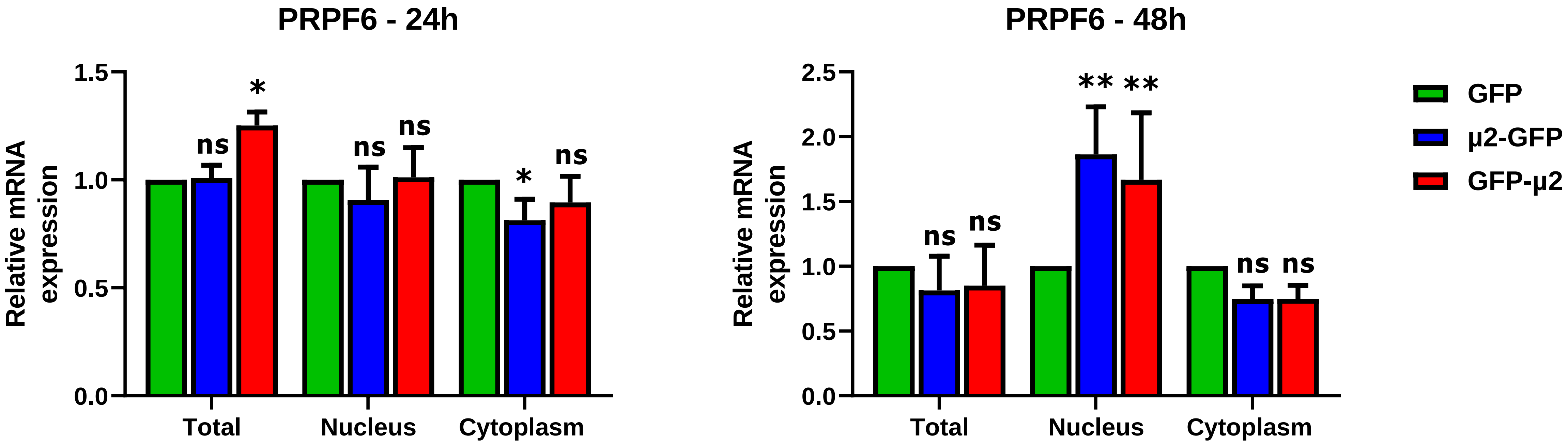
PRPF6 - 24h
Relative mRNA expression
1.5
1.0
0.5
0.0
ns
*
ns
ns
*
ns
Total
Nucleus
Cytoplasm
PRPF6 - 48h
Relative mRNA expression
2.5
2.0
1.5
1.0
0.5
0.0
ns
ns
**
**
ns
ns
Total
Nucleus
Cytoplasm
GFP
μ2-GFP
GFP-μ2

Figure S5

Figure S6. Validation of the polysomal fractionation by automated chip-based microcapillary electrophoresis. RNA was harvested from 30% of each fraction, and subjected to microcapillary electrophoresis using a LabChip GX Touch HT Nucleic Acid Analyzer (PerkinElmer). The electrophoregrams confirmed an absence of both 18s and 28s ribosomal RNA from the free fraction, only the 18s ribosomal RNA for the 40s fraction, and both rRNA for the 80s, P1, P2, and P3 fractions. The 60s present a small amount of 18s rRNA, which is consistent with the peak of 80s eluting very close to the large subunit of the ribosome. A representative result is shown.

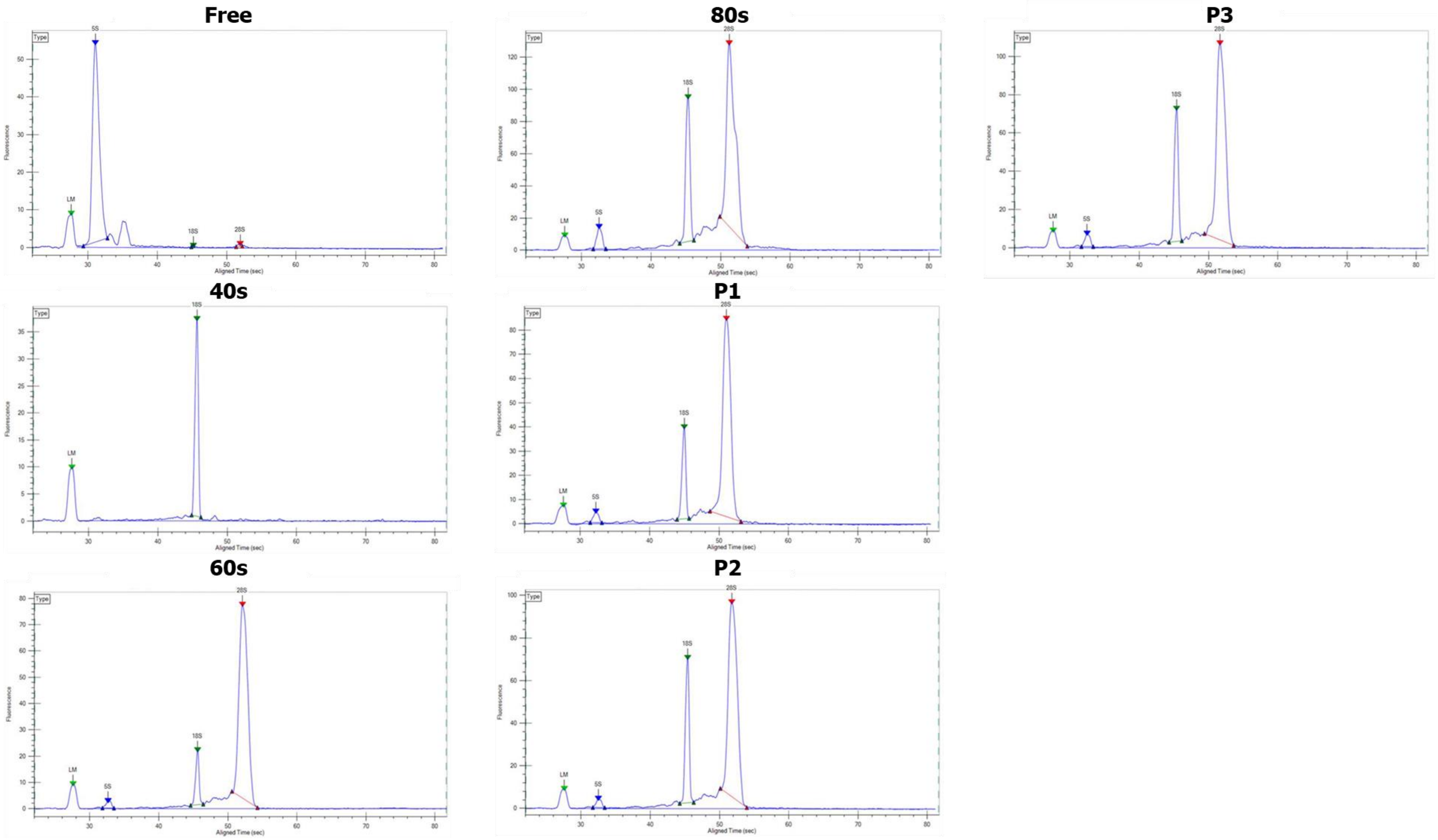
Free
80s
P3
40s
P1
60s
P2
Type
LM
5S
18S
28S
Fluorescence
Aligned Time (sec)

Figure S6

Figure S7. Relative mRNA level determined by qPCR for B2M and SNRPA in 40S+60S, 80S, and polysomal (P1+P2+P3) fractions from GFP or µ2-GFP expressing cells at 48 h post-transfection. Lysates were prepared, separated on a 5-50% sucrose gradient, and 30% of each fraction was subjected to RNA extraction using Qiazol. RNA was reverse transcribed using a fixed volume for each sample and subjected to qPCR with U6 snRNA as the housekeeping gene, as it was stable between all fractions. The relative expression is calculated against the first sample in the GFP condition for the 40S+60S fraction. n=3, biological replicates, two-way ANOVA with Dunnett's multiple comparisons test against the GFP control for each fraction (ns, $P>0.05$; *, $P\leq0.05$).

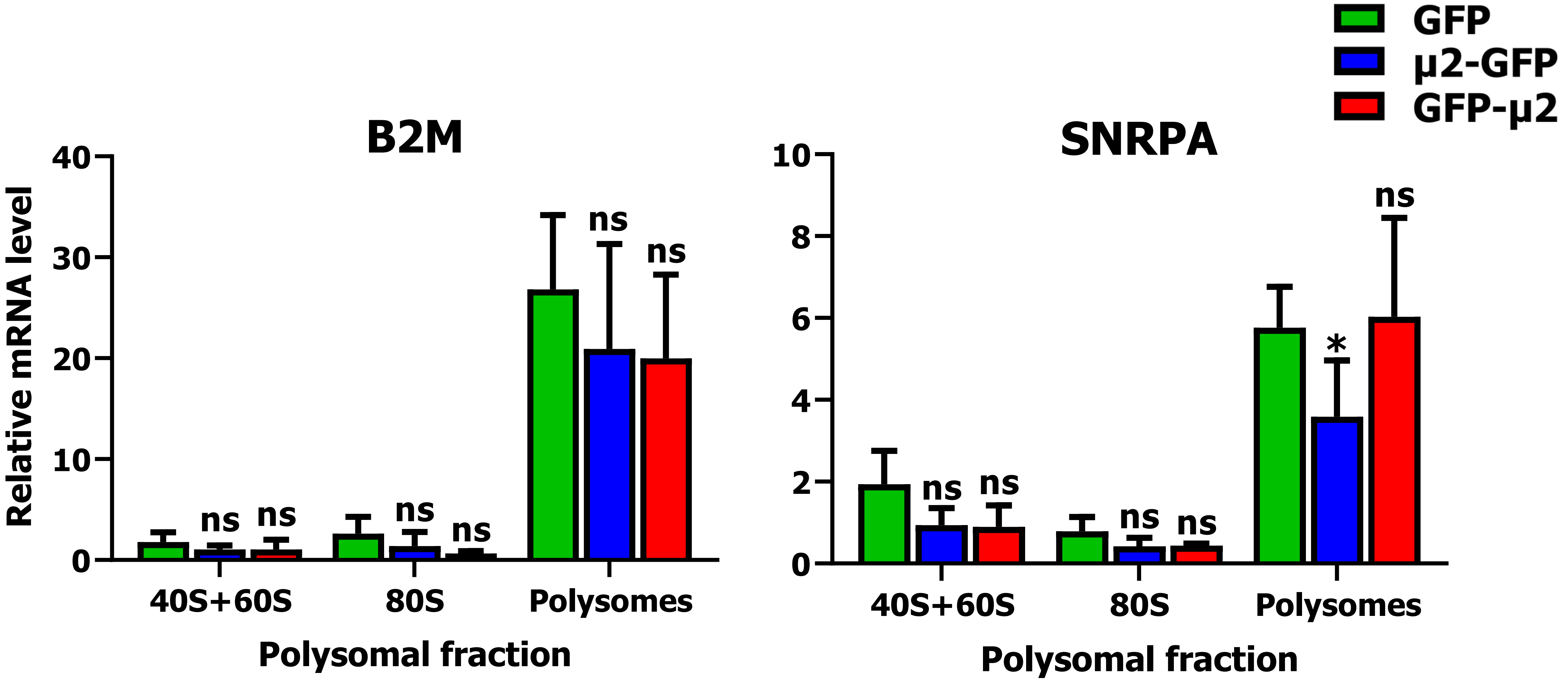
GFP
μ2-GFP
GFP-μ2
B2M
Relative mRNA level
40
30
20
10
0
ns
ns
ns
ns
ns
ns
40S+60S
80S
Polysomes
Polysomal fraction
SNRPA
10
8
6
4
2
0
ns
ns
ns
ns
*
ns
40S+60S
80S
Polysomes
Polysomal fraction

Figure S7

Figure S8. Schematic of the collection of the polysomal fractions from the sucrose gradients. Grey area were sent to waste, and blue section were collected. The name of the fractions is noted on the top of the profile. The number of ribosome present on the mRNA is also noted right above the peaks.

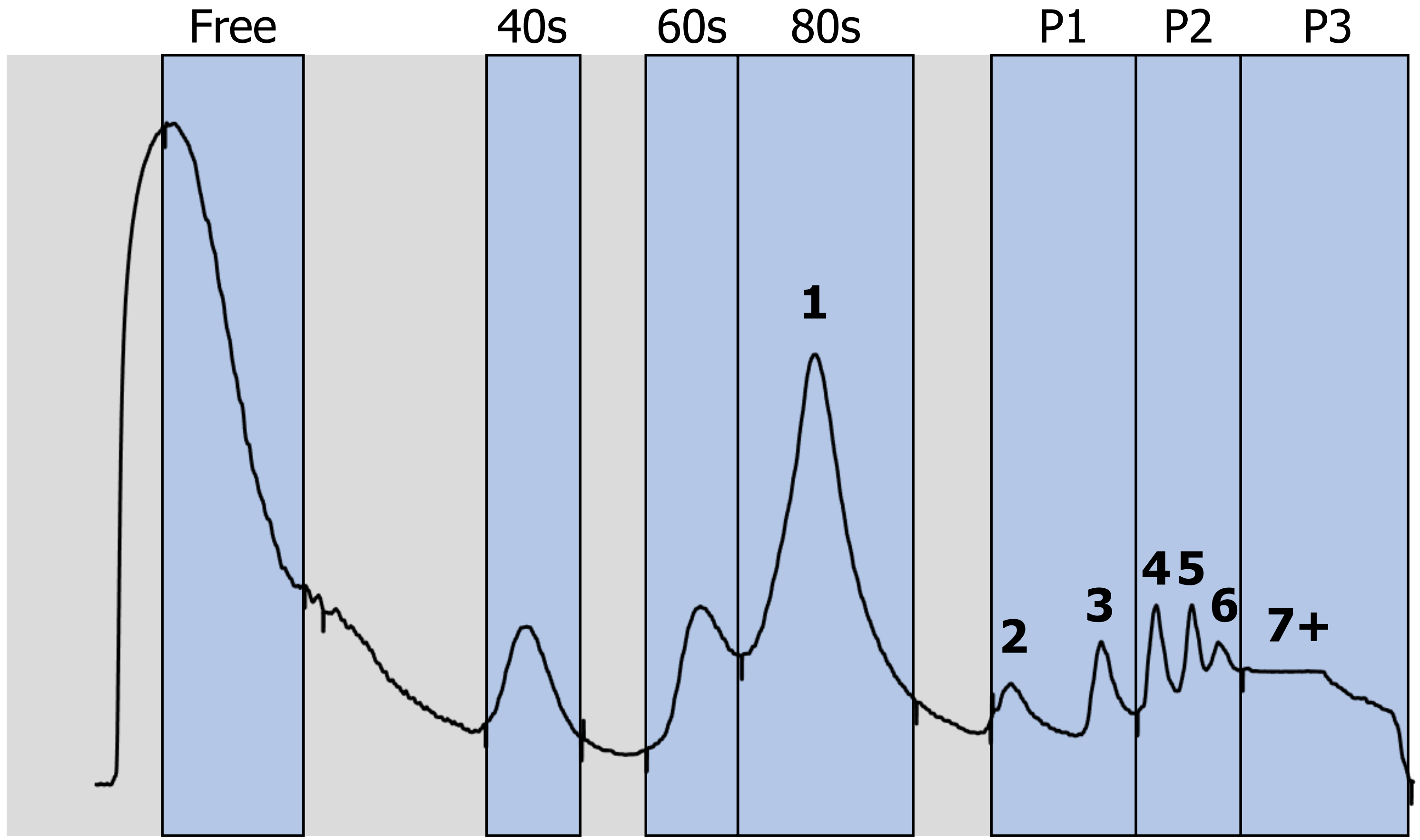

Figure S8
